# KIR3DL1-Negative CD8 T Cells and KIR3DL1-Negative Natural Killer Cells Contribute to the Advantageous Control of Early Human Immunodeficiency Virus Type 1 Infection in *HLA-B Bw4* Homozygous Individuals

**DOI:** 10.3389/fimmu.2018.01855

**Published:** 2018-08-10

**Authors:** Xin Zhang, Xiaofan Lu, Christiane Moog, Lin Yuan, Zhiying Liu, Zhen Li, Wei Xia, Yuefang Zhou, Hao Wu, Tong Zhang, Bin Su

**Affiliations:** ^1^Center for Infectious Diseases, Beijing You’an Hospital, Capital Medical University, Beijing, China; ^2^Beijing Key Laboratory for HIV/AIDS Research, Beijing, China; ^3^INSERM U1109, Fédération Hospitalo-Universitaire (FHU) OMICARE, Fédération de Médecine Translationnelle de Strasbourg (FMTS), Université de Strasbourg, Strasbourg, France; ^4^Vaccine Research Institute (VRI), Créteil, France

**Keywords:** human immunodeficiency virus type 1, immunity, KIR3DL1 receptor, acute/early infection, *Bw4* homozygotes

## Abstract

*Bw4* homozygosity in human leukocyte antigen class B alleles has been associated with a delayed acquired immunodeficiency syndrome (AIDS) development and better control of human immunodeficiency virus type 1 (HIV-1) viral load (VL) than *Bw6* homozygosity. Efficient CD8 T cell and natural killer (NK) cell functions have been described to restrain HIV-1 replication. However, the role of KIR3DL1 expression on these cells was not assessed in *Bw4*-homozygous participants infected with HIV-1 CRF01_A/E subtype, currently the most prevalent subtype in China. Here, we found that the frequency of KIR3DL1-expressing CD8 T cells of individuals homozygous for *Bw6* [1.53% (0–4.56%)] was associated with a higher VL set point (Spearman *r*_s_ = 0.59, *P* = 0.019), but this frequency of KIR3DL1^+^CD8^+^ T cells [1.37% (0.04–6.14%)] was inversely correlated with CD4 T-cell count in individuals homozygous for *Bw4* (*r*_s_ = −0.59, *P* = 0.011). Moreover, CD69 and Ki67 were more frequently expressed in KIR3DL1^−^CD8^+^ T cells in individuals homozygous for *Bw4* than *Bw6* (*P* = 0.046 for CD69; *P* = 0.044 for Ki67), although these molecules were less frequently expressed in KIR3DL1^+^CD8^+^ T cells than in KIR3DL1^−^CD8^+^ T cells in both groups (all *P* < 0.05). KIR3DL1^−^CD8^+^ T cells have stronger p24-specific CD8^+^ T-cell responses secreting IFN-γ and CD107a than KIR3DL1^+^CD8^+^ T cells in both groups (all *P* < 0.05). Thus, KIR3DL1 expression on CD8 T cells were associated with the loss of multiple functions. Interestingly, CD69^+^NK cells lacking KIR3DL1 expression were inversely correlated with HIV-1 VL set point in *Bw4*-homozygous individuals (*r*_s_ = −0.52, *P* = 0.035). Therefore, KIR3DL1^−^CD8^+^ T cells with strong early activation and proliferation may, together with KIR3DL1^−^CD69^+^NK cells, play a protective role during acute/early HIV infection in individuals homozygous for *Bw4*. These findings highlight the superior functions of KIR3DL1^−^CD8^+^ T cells and KIR3DL1^−^CD69^+^NK cells being a potential factor contributing to delayed disease progression in the early stages of HIV-1 infection.

## Introduction

CD8 T cells and natural killer (NK) cells contribute to the host immune response to human immunodeficiency virus (HIV) infection, but the functions of these cells can be repressed by the inhibitory molecules on their surface. The principal NK cell receptors are natural cytotoxicity receptors, C-type lectin-like receptors, and killer cell immunoglobulin-like receptors (KIRs). Of these molecules, natural cytotoxicity receptors are the most specific NK cell marker. C-type lectin-like receptors and KIRs are also expressed on CD8 T lymphocytes (1–3). The KIR3DL1 receptor, a member of the KIR family, interacts with its ligand to transmit inhibitory signals that suppress the NK cell-mediated lysis of target cells *via* cytoplasmic immunoreceptor tyrosine-based inhibitory motifs (ITIMs). KIR3DL1 recognizes the Bw4 motif on human leukocyte antigen (HLA) class B molecules, which may be classified as Bw4 or Bw6 allotypes, according to the serological epitopes spanning residues 77–83 on the α1-helix of the HLA-I molecule (4).

The CD8 T cells that can be activated to induce anti-HIV-1-specific responses are restricted by HLA antigens, including HLA-B alleles in particular, which play a much greater role in mediating antiviral cytotoxic T-lymphocyte (CTL) responses than HLA-A and HLA-C alleles (5, 6). The HLA-B*27 and -B*57 alleles, both of which carry the Bw4 motif, are associated with low HIV-1 viremia and slower progression to acquired immunodeficiency syndrome (AIDS). HLA-B*44 and -B*51 have not consistently been shown to play a protective role in HIV-1 infection, and the HLA-B*05, -B*13, -B*17, -B*37, and -B*38 alleles and some other non-protective HLA antigens, also express the Bw4 public motif. Other alleles, such as HLA-B*07, -B*08, -B*14, -B*35, -B*40, -B*41, -B*53, -B*56, carry the Bw6 motif. The HLA-B*08, -B*35, -B*53, -B*55, and -B*56 alleles are associated with rapid progression to AIDS (7). *HLA-Bw4* homozygosity is associated with a lower risk of HIV transmission (8), better control of HIV-1 viremia and protection against AIDS (9, 10) whereas *HLA-Bw6* homozygosity accelerates HIV-1 disease progression (11, 12), but the precise mechanisms underlying this protection remain unknown.

KIRs, some inhibitory and others activating, are expressed on the surface of a subpopulation of CD8 T cells with a memory and effector phenotype (13). KIR expression is relatively stable on NK cells, and the frequency of KIR-positive CD8 T cells increases with age, mostly due to the accumulation of terminally differentiated T cells (14). KIR-positive CD8 T cells are particularly abundant in participants with HIV-1 (15) or cytomegalovirus (CMV) (16) infection. By contrast, very few HIV-specific, CMV-specific CD8 T cells (17–19) in HIV-1-infected or healthy individuals express KIR receptors, including KIR3DL1. It has, therefore, been suggested that KIR3DL1-positive CD8 T cells function poorly in HIV-1-infected individuals displaying homozygosity for *Bw4*. By contrast, *Bw4* homozygosity may strengthen the functions of KIR3DL1-negative CD8 T cells, resulting in enhanced immune surveillance and playing a predominant role in protection against HIV-1 infection. Besides, KIR3DL1-expressing NK cells can play its role through the ligand of Bw4 motif *via* a known process of NK cell licensing, but it was not uncertain whether NK cells especially KIR3DL1-negative NK cells activity from *HLA-Bw4* homozygous individuals were helpful for restraining HIV-1 replication compared with *HLA-Bw6* homozygous carriers.

In this study, we observed, in the Beijing PRIMO prospective acute HIV-1 infection cohort, early activation, proliferation capacity, and the HIV-1-specific responses of KIR3DL1-positive CD8 T cells were significantly weaker than those of KIR3DL1-negative CD8 T cells in individuals homozygous for *Bw4*. More interestingly, KIR3DL1-negative NK cell activation capacity was negatively related to the viral load (VL) set point and the number of HIV-1-specific KIR3DL1^−^CD8^+^ T cells responses in individuals homozygous for *Bw4* during acute/early HIV-1 infection. These findings improve our understanding of KIR-mediated control and CD8 T/NK-cell response mechanisms in primary HIV infection.

## Materials and Methods

### Study Subjects

The study subjects were recruited from the Beijing PRIMO clinical cohort, a prospective study cohort of HIV-1-negative men who have sex with men (MSM) designed to identify cases of acute HIV-1 infection at Beijing You’an Hospital, Beijing, China, which has been running since October 2006. The enrolled participants were monitored every 2 months for HIV antibodies, HIV RNA levels, and clinical signs of acute/early infection, as previously described (20). The progression of early HIV-1 infection can be depicted as six discrete stages, as proposed by Fiebig et al. (21). In total, 17 of 24 participants homozygous for *Bw4* and 17 of 40 participants homozygous for *Bw6* were between the Fiebig stage VI and 6 months after infection, all in acute/early stages of HIV infection, and without antiretroviral therapy (ART), were enrolled in this study. These 34 participants were infected with a circulating recombinant form CRF01_A/E subtype based on *pol* sequence (22–24), currently the most prevalent subtype in China, as shown by our results for the Beijing PRIMO cohort (23, 25, 26). The opportunistic infections, tuberculosis, autoimmune diseases, or HBV/HCV co-infection were excluded and this exclusion criteria was displayed in the flow chart (Figure S1 in Supplementary Material). These enrolled participants were followed up for 3 years. During follow-up, we recorded whether CD4 T-cell count fell below 350/μl for three consecutive measurements, the first date of measurement being fixed as the time at which CD4 count dropped below 350/μl. CD4 T-cell count did not subsequently rise above 350/μl before the initiation of ART or remained below 350/μl after the initiation of ART (10). Blood samples were collected, and peripheral blood mononuclear cells (PBMCs) and plasma were isolated and cryopreserved. We enrolled 33 age-matched HIV-1-negative individuals from the MSM population with high-risk behaviors as controls.

### HLA Class I Allele Genotyping

Genotypes including those for *HLA-A, HLA-B*, and *HLA-C* were determined by sequence-specific primer (SSP)-PCR (at the Weatherall Institute of Molecular Medicine, John Radcliffe Hospital, Oxford University, Oxford, UK). Genomic DNA was extracted from PBMCs with the QIAamp DNA Blood MiniKit (Qiagen, Valencia, CA, USA) according to the manufacturer’s instructions. The Bw4 and Bw6 motifs of the *HLA-B* alleles were identified by SSP-PCR, as previously described (10).

### Cell Staining and Flow Cytometry Analysis

**Figure 1:**
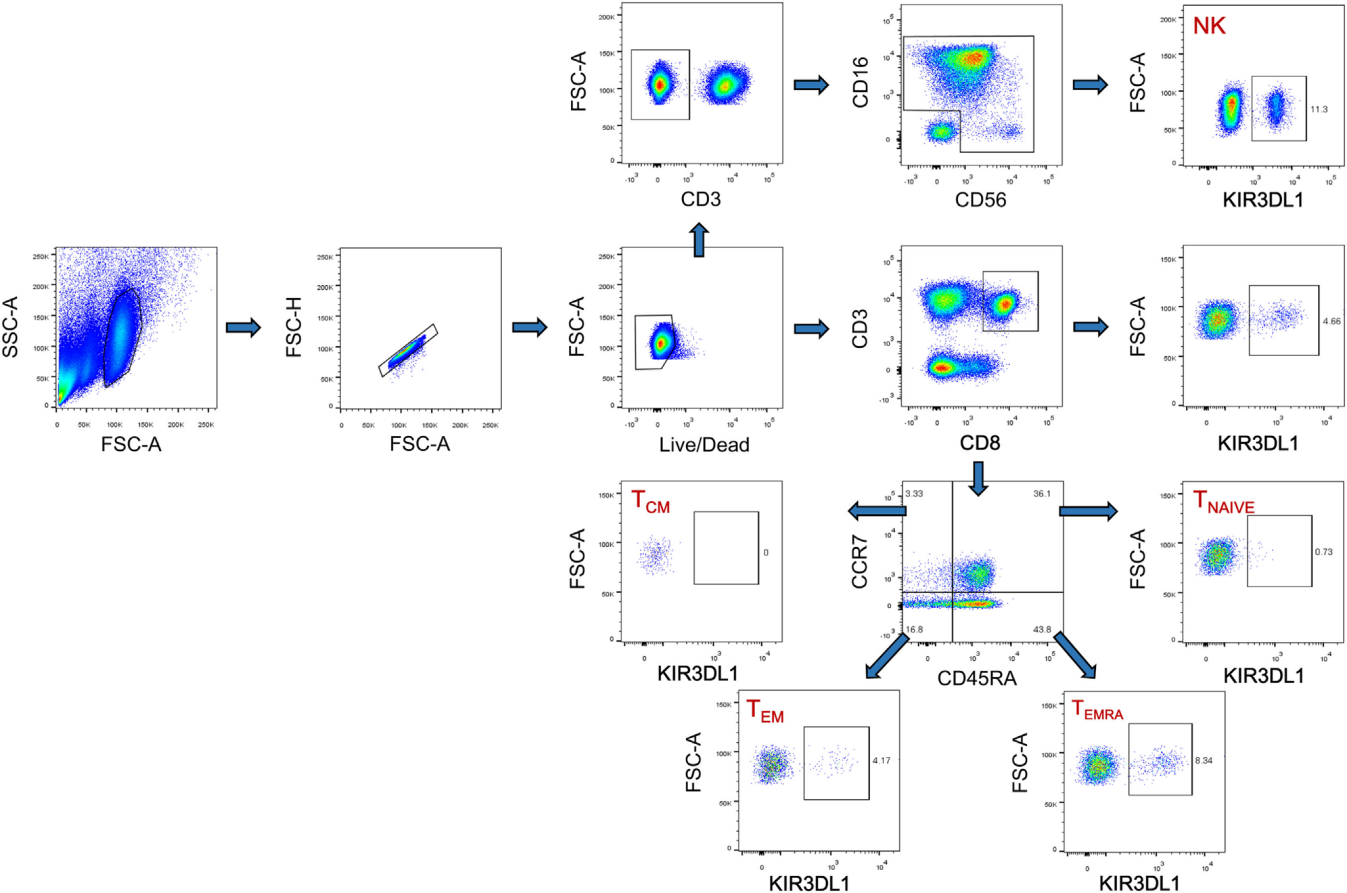
The gating strategy for flow cytometric analysis of KIR3DL1 expressing on CD8 T cells and NK cells. Among all events, forward angle and SSC light gating were gated on lymphocytes and were used to exclude cell debris from the analysis. Forward height and forward area were used to exclude doublet cells, and cells were labeled with Live/Dead fixable viability stain 510, and dead cells were excluded. Then CD3^+^CD8^+^ T cells, KIR3DL1^+^CD8^+^ T cells, CD8^+^ T cell subsets (T_NAIVE_, T_CM_, T_EM_, and T_EMRA_), and KIR3DL1 expressing on different CD8^+^ T cell subsets were gated; CD3^−^ cells, CD16^+^CD56^+^ NK cells, and KIR3DL1-expressing on NK cells were also analyzed simultaneously. The final analysis was performed with FlowJo software, which generated a graphical output. FSC, forward scatter; SSC, side scatter; NK, natural killer.

Cryopreserved PBMCs were thawed in RPMI 1640 medium (Hyclone, Logan, UT, USA) supplemented with 10% fetal bovine serum (Hyclone), 50 IU/ml penicillin–streptomycin (Hyclone), and 2 mM l-glutamine (Hyclone). They were then stained with fluorescence-conjugated human monoclonal antibodies (mAbs) including APC-CD3 (clone HIT3a; BioLegend, San Diego, CA, USA), PE-CD8 (clone IM0452U; Beckman Coulter, Brea, CA, USA), APC-Cy7-CD69 (clone FN50; BioLegend), Percp-Cy5.5-KIR3DL1 (clone DX9; BioLegend), FITC-CCR7 (clone G043H7; BioLegend), and Pacific blue-CD45RA (clone HI100; BioLegend). The PBMCs were then fixed and permeabilized (Cat. No: 00-5523-00; eBiosciences, San Diego, CA, USA) and were subjected to intracellular staining with PE-Cy7-Ki67 antibodies (cloneKi-67; BioLegend). NK cells were stained with a panel of NK cell-specific antibodies including PE-Cy7-CD3 (clone HIT3a; BioLegend), FITC-CD16 (clone 3G8; BioLegend), PE-CD56 (clone HCD56; BioLegend), and Percp-Cy5.5-KIR3DL1 (clone DX9; BioLegend), APC-Cy7-CD69 (clone FN50; BioLegend). The isotype control mAbs were purchased from the corresponding companies. Cytometer setup and tracking calibration particles were used to ensure that fluorescence intensity measurements were consistent in all experiments. Flow cytometry Comp-Beads kits (BD Bioscience, San Jose, CA, USA) were used for compensation. Gating on forward scatter and side scatter light was used to exclude cell debris from the analysis; forward height and forward area were used to exclude doublet cells, and dead cells were excluded by staining with Live/Dead fixable viability stain 510 (BD Biosciences, San Jose, CA, USA). At least 200,000 PBMCs were acquired with a BD CantoII flow cytometer, as previously described (27, 28), and the data were analyzed with Flowjo Software version 10.0 (Treestar, Ashland, OR, USA). The strategies for the analysis of flow cytometry data are detailed in Figure 1.

### Intracellular Cytokine Staining and Cell Degranulation Staining Assays

Thawed and incubated overnight PBMCs from participants infected with subtype CRF01_A/E virus were stimulated with 2 µg/ml pooled CRF01_A/E p24 peptides, 1 µg/ml purified antibodies against CD28/CD49d (Cat. No. 347690; BD Biosciences, San Jose, CA, USA) and PE-anti-CD107a antibody (clone H4A3; BioLegend). After 1 h, 3 µg/ml brefeldin A and 2 µM monensin agents (eBioscience™ 1,000×) were added to the cells, which were incubated for 5 h. Control cells were stimulated with 1 µg/ml purified anti-CD28/CD49d antibody in the absence of peptide. Positive control cells were stimulated with 20 ng/ml phorbol 12-myristate 13-acetate (Sigma-Aldrich, St. Louis, MO, USA) and 1 µg/ml ionomycin (Sigma), and cultured for 6 h at 37°C. The PBMCs were then stained with PE-Cy7-CD3 (clone HIT3a; BioLegend), FITC-CD8 (clone IM0451U; Beckman), APC-CD69 (clone FN50; BioLegend), and Percp-Cy5.5-KIR3DL1 (clone DX9; BioLegend) antibodies for 20 min at room temperature. The PBMCs were fixed and permeabilized with BD FACS™ permeabilizing solution (Cat. No. 340457), and intracellular staining was performed for interferon gamma (IFN-γ) (Brilliant Violet 421™-conjugated; Cat. No. 502532; BioLegend) for 30 min at 4°C. The cells were then analyzed in a BD CantoII flow cytometer as described above.

### Detection of IFN-γ-Producing Cells in Enzyme-Linked Immunosorbent Spot (ELISPOT) Assays

Frozen PBMCs from participants infected with HIV-1 subtype CRF01_A/E were thawed and incubated overnight at 37°C under an atmosphere containing 5% CO_2_. PBMCs were pulsed with 2 µg/ml CRF01_A/E p24 peptides (same as above) for 18–24 h. The peptides used were 18 amino acids long and overlapped by 10 amino acids (Table S1 in Supplementary Material). 5 µg/ml phytohemagglutinin was used as experimental positive control and 2 µg/ml EBV/Flu/CMV (EFC) peptides were used as quality control (29); negative control was used with RPMI 1640 medium. HIV-1-specific CD8 T-cell responses were measured by quantifying IFN-γ release with an ELISPOT assay (30), using the anti-IFN-γ mAb 1-D1K (Mabtech AB, Nacka, Sweden), the biotinylated anti-IFN-γ mAb 7-B6-1 (Mabtech AB), and streptavidin-alkaline phosphatase conjugate (Mabtech AB). IFN-γ-producing cells were counted with an ELISPOT reader (Antai Yongxin Medical Technology, Beijing, China), and the results are expressed as the number of spot-forming cells per million PBMCs. ELISPOT results were shown in Figure S2 in Supplementary Material. Results were considered positive only if there were more than 50 spot-forming cells/million PBMCs and if there were at least three times as many spot-forming cells than in the negative control as reported in our previous study (31).

### CD4 T-Cell Count and VL Measurement

Routine blood CD4 T-cell counts (cells/μl) were measured by four-color flow cytometry with human CD45^+^, CD3^+^, CD4^+^, and CD8^+^ cell markers (BD Biosciences), on peripheral whole-blood samples from each patient, in FACS lysing solution (BD Biosciences), according to the manufacturer’s instructions. Plasma HIV-1 VL (copies/ml of plasma) was quantified by real-time PCR (Abbott Molecular Inc., Des Plaines, IL, USA). This assay has a sensitivity of 40 copies/ml of plasma for viral RNA detection. The VL set point at the very early stage of HIV-1 infection was calculated and reported in our previous study (20).

### Statistical Analysis

Data are expressed as mean ± SD. Statistical analysis was performed with GraphPad Prism software version 5.03 (GraphPad Software, San Diego, CA, USA). Differences were analyzed in Student’s *t*-tests (unpaired *t*-test for unpaired variables and paired *t*-test for paired variables) or non-parametric Mann–Whitney *U* tests for non-parametric samples. Spearman’s rank correlation coefficient, denoted as *r*_s_, is a statistical value that measures the monotonic relationship between two variables. Differences were considered statistically significant if *P* < 0.05 in two-tailed tests. The detailed statistical analysis is described in the figure legends.

## Results

### Demographics of Individuals in the Acute/Early Phase of HIV-1 Infection

*Bw4* homozygosity has been reported to be associated with the control of HIV-1 viremia and protection against AIDS, and with a less marked decline in CD4 T-cell counts in HIV-1-infected individuals (9). We investigated the early effects of KIR3DL1 expression on CD8 T cells in individuals homozygous for the *Bw4* or *Bw6* genotype included during the acute/early phase of HIV-1 infection. The demographic features of these individuals are described in Table 1. VL set point was significantly lower in individuals homozygous for *Bw4* than in individuals homozygous for *Bw6* (*P* = 0.002, Table 1), whereas CD4 T-cell count appeared to trend higher in individuals homozygous for *Bw4*, although it was not statistically significant (*P* = 0.083, Table 1). Furthermore, CD4 T-cell count fell below 350/μl during the first 3 years of HIV-1 infection more frequently in individuals homozygous for *Bw6* than in individuals homozygous for *Bw4* (*P* = 0.013). KIR3DL1 expression and the functions of KIR3DL1^+^CD8^+^ and KIR3DL1^−^CD8^+^ T cells were investigated, to explore the effects of KIR3DL1 expression on CD8 T cells in the presence and absence of *Bw4* homozygosity.

**Table 1:** Demographics of HIV-1-infected individuals.

	Age	VL set point, Log_10_ (copies/ml)	CD4 T-cell count (cells/μl)	HLA-*B alleles	VL at 3 years after infection, Log_10_ (copies/ml)	CD4 decline to <350 within 3 years
**Bw4/4**						
1	34	4.37	374	B*37, B*51	4.29	Y
2	23	3.84	1,058	B*51, B*51	4.54	N
3	25	2.94	919	B*57, B*27	3.10	N
4	26	5.19	117	B*58, B*51	ND^a^	Y
5	27	4.43	602	B*13, B*27	5.06	N
6	28	3.87	455	B*13, B*13	ND^a^	Y
7	47	4.04	561	B*13, B*13	4.43	N
8	26	3.62	820	B*37, B*58	5.02	Y
9	24	3.41	668	B*44, B*52	4.04	N
10	41	3.68	345	B*27, B*52	3.94	Y
11	24	2.50	345	B*44, B*58	3.75	N
12	22	4.82	546	B*44, B*38	5.02	Y
13	21	3.09	505	B*44, B*57	3.23	N
14	46	2.81	505	B*44, B*13	3.07	N
15	47	3.85	498	B*44, B*13	4.77	N
16	34	2.95	933	B*52, B*52	3.21	N
17	31	3.94	530	B*44, B*13	1.88^a^	N
Mean ± SD	30.9 ± 9.0	3.73 ± 0.73	575 ± 242.2			

**Bw6/6**						
18	39	4.24	358	B*15, B*15	3.65	Y
19	42	4.90	222	B*15, B*35	3.71^a^	Y
20	56	4.40	513	B*08, B*54	4.39	Y
21	25	3.27	827	B*39, B*15	3.82	N
22	24	3.40	303	B*40, B*67	4.67	Y
23	27	4.66	899	B*15, B*67	4.17	Y
24	38	5.24	258	B*40, B*15	5.16	N
25	39	4.30	196	B*40, B*15	4.07	Y
26	39	4.04	454	B*07, B*15	3.95	N
27	49	5.07	281	B*08, B*15	4.84	Y
28	25	4.88	351	B*46, B*15	4.35	Y
29	23	4.85	205	B*55, B*15	ND^a^	Y
30	36	5.23	554	B*40, B*46	5.28	Y
31	34	4.60	516	B*48, B*40	5.40	Y
32	27	4.42	770	B*08, B*45	4.18	Y
33	54	4.30	308	B*07, B*35	NA	–^b^
34	25	4.46	353	B*07, B*48	3.15^a^	Y
Mean ± SD	35.4 ± 10.6	4.48 ± 0.56	433 ± 219.6	–	–	
*P* value	0.195^c^	0.002^d^	0.083^e^		0.183^f^	0.013^g^

*^a^Patients initiating ART*.

*^b^This patient had less than 3 years of follow-up visits after HIV-1 infection*.

*The significance of the difference in age^c^, VL set point^d^, CD4 T-cell count^e^, VL at 3 years after infection^f^ (patients initiating ART were excluded) between the two groups of individuals was assessed in Student’s *t*-test. Differences were considered statistically significant at *P* < 0.05*.

*^g^Fisher’s exact test*.

*Y, yes; N, no; NA, not applicable; ND, not detected; HIV-1, human immunodeficiency virus type 1; HLA, human leukocyte antigen; ART, antiretroviral therapy; VL, viral load*.

### Higher Percentage of KIR3DL1 Expression on CD8 T_EMRA_ Cells Was Correlated With HIV-1 VL Set Point in *Bw6*-Homozygous Individuals

**Figure 2:**
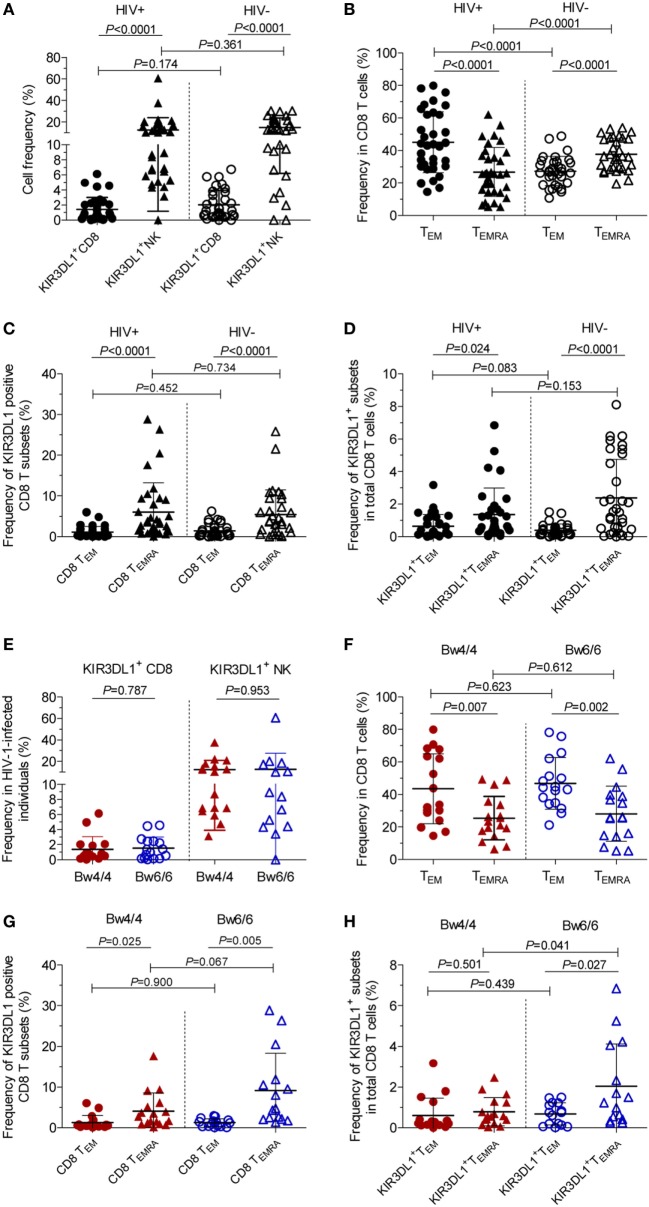
KIR3DL1 expression on natural killer (NK) cells, total CD8 T cells, and CD8 T-cell subsets. Differences in KIR3DL1 expression on total CD8 T cells or NK cells **(A)**, CD8 T_EM_ (CD45RA^−^CCR7^−^) and CD8 T_EMRA_ (CD45RA^+^CCR7^−^) subsets from total CD8 T cells **(B,C)**, and KIR3DL1^+^ T_EM_ and KIR3DL1^+^ T_EMRA_ from total CD8 T cells between human immunodeficiency virus type 1 (HIV-1)-positive patients at the acute/early stage and HIV-1-negative individuals **(D)**. KIR3DL1 expression on CD8 T cells or NK cells **(E)**, CD8 T_EM_ and CD8 T_EMRA_ subsets **(F,G)**, and KIR3DL1^+^ T_EM_ and KIR3DL1^+^ T_EMRA_ from total CD8 T cells **(H)** in HIV-1-infected patients homozygous for *Bw4* or *Bw6*. Data are expressed as mean ± SD. The significance of differences was analyzed in unpaired *t*-tests, with *P* < 0.05 considered significant. Bw4/4: *Bw4* homozygotes (red); Bw6/6: *Bw6* homozygotes (blue).

Figure 1 displayed the flow cytometric gating strategies for the analysis of KIR3DL1 expression on different CD8 T cells and NK cells. In HIV-1-infected individuals, the percentages of KIR3DL1^+^CD8^+^ T cells and KIR3DL1^+^NK cells were 1.49% (0–6.14%) and 12.64% (0–60.6%), respectively, versus 2.03% (0–6.74%) and 14.96% (0–30.18%) in individuals negative for HIV-1 antibody, and this difference was not statistically significant, as shown in Figure 2A. The percentage of CD8 T_EM_ (CD45RA^−^CCR7^−^, effector memory) cells was significantly higher than that of CD8 T_EMRA_ cells (CD45RA^+^CCR7^−^, terminally differentiated effector memory) in HIV-1-infected individuals (Figure 2B). KIR3DL1 was expressed principally on the cells of the CD8 T_EMRA_ subset, and also on those of the CD8 T_EM_ subset, as shown in Figure 2C. The percentage of KIR3DL1^+^CD8^+^ T_EM_ cells in the total CD8 T-cell population was much lower than that of KIR3DL1^+^CD8^+^ T_EMRA_ cells (*P* < 0.05), in both HIV-1-positive and HIV-1-negative individuals (Figure 2D). Furthermore, the frequency of KIR3DL1^+^CD8^+^ T cells in *Bw4*-homozygous individuals was similar to that in *Bw6*-homozygous individuals [1.37% (0.04–6.14%) versus 1.53% (0–4.56%)]. The percentage of KIR3DL1^+^NK cells was 12.5% (3.15–37.67%) in individuals homozygous for *Bw4* and 12.76% (0–60.60%) in individuals homozygous for *Bw6*, as shown in Figure 2E. KIR3DL1 expression on NK and CD8 T cells was independent of homozygosity for *Bw4* or *Bw6* in *HLA-B* alleles (Figure 2E). The percentage of CD8 T_EM_ cells was higher than that of CD8 T_EMRA_ cells in both individuals homozygous for *Bw4* and those homozygous for *Bw6* (Figure 2F). KIR3DL1 was more frequently expressed on CD8 T_EMRA_ cells than on CD8 T_EM_ cells and was independent of *HLA-B* locus-specific Bw4 or Bw6 motifs (Figure 2G). In individuals homozygous for *Bw6*, KIR3DL1^+^CD8^+^ T_EMRA_ cells had a higher frequency than KIR3DL1^+^CD8^+^ T_EM_ cells among total CD8 T cells (*P* = 0.027, Figure 2H). In addition, the percentage of KIR3DL1^+^CD8^+^ T_EMRA_ cells among total CD8 T cells was higher in HIV-1-infected individuals homozygous for *Bw6* than in those homozygous for *Bw4* (*P* = 0.041, Figure 2H).

**Figure 3:**
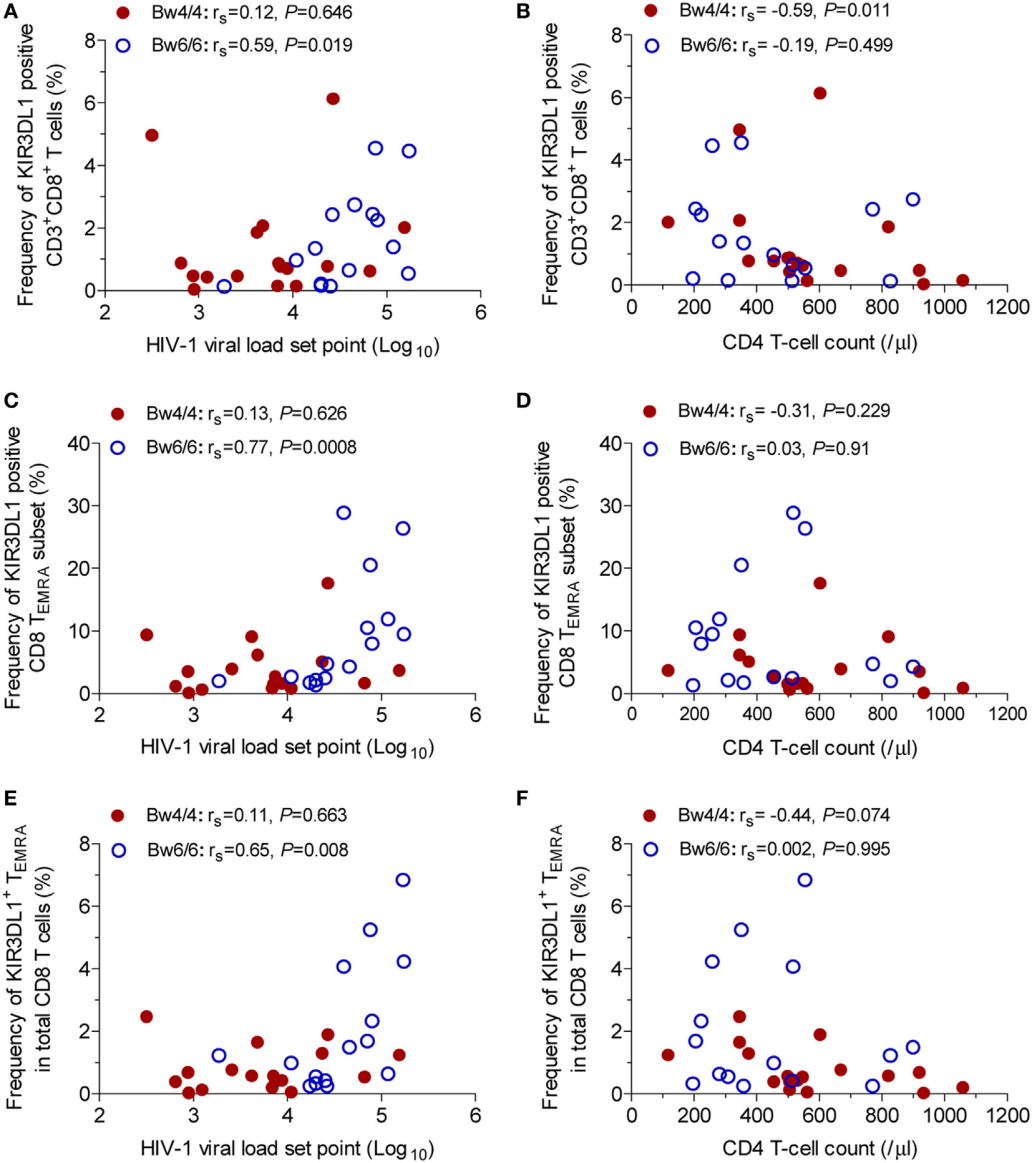
Correlation between KIR3DL1 expression on CD8 T cells with human immunodeficiency virus type 1 (HIV-1) viral load (VL) set point and CD4 T-cell count in individuals with different *HLA-B* serological genotypes. Correlation of KIR3DL1 expression on CD8 T cells with HIV-1 VL set point **(A)** and CD4 T-cell count **(B)**, of the percentage of CD8 T_EMRA_ cells expressing KIR3DL1 with HIV-1 VL set point **(C)** and CD4 T-cell count **(D)**, and of KIR3DL1^+^ T_EMRA_ levels as a proportion of total CD8 T cells with HIV-1 VL set point **(E)** and CD4 T-cell count **(F)**. Correlations between two variables were analyzed in non-parametric Spearman’s rank correlation tests, with *P* < 0.05 considered significant.

KIR3DL1^+^CD8^+^ T-cell percentage and VL set point were positively correlated in *Bw6*-homozygous individuals (*r*_s_ = 0.59, *P* = 0.019, Figure 3A), but not in *Bw4*-homozygous individuals (*r*_s_ = 0.12, *P* = 0.646, Figure 3A). Conversely, the inverse association between KIR3DL1^+^CD8^+^ T-cell levels and CD4 T-cell count was observed only in individuals homozygous for *Bw4* (*r*_s_ = −0.59, *P* = 0.011, Figure 3B). In individuals homozygous for *Bw6*, the percentage of CD8 T_EMRA_ cells expressing KIR3DL1 was positively correlated with VL set point (*r*_s_ = 0.77, *P* = 0.0008, Figure 3C), but not with CD4 T-cell count (Figure 3D).

In addition, a positive association between the percentage of KIR3DL1^+^T_EMRA_ cells among total CD8 T cells and HIV-1 VL set point was observed in individuals homozygous for *Bw6* (*r*_s_ = 0.65, *P* = 0.008, Figure 3E), whereas a trend toward a negative relationship between KIR3DL1^+^T_EMRA_ levels and CD4 T-cell count was observed in individuals homozygous for *Bw4* (*r*_s_ = −0.44, *P* = 0.074, Figure 3F).

### Higher Frequencies of KIR3DL1^−^CD69^+^CD8^+^ T Cells and KIR3DL1^−^Ki67^+^CD8^+^ T Cells Were Associated With Homozygosity for *Bw4* in *HLA-B* Alleles

**Figure 4:**
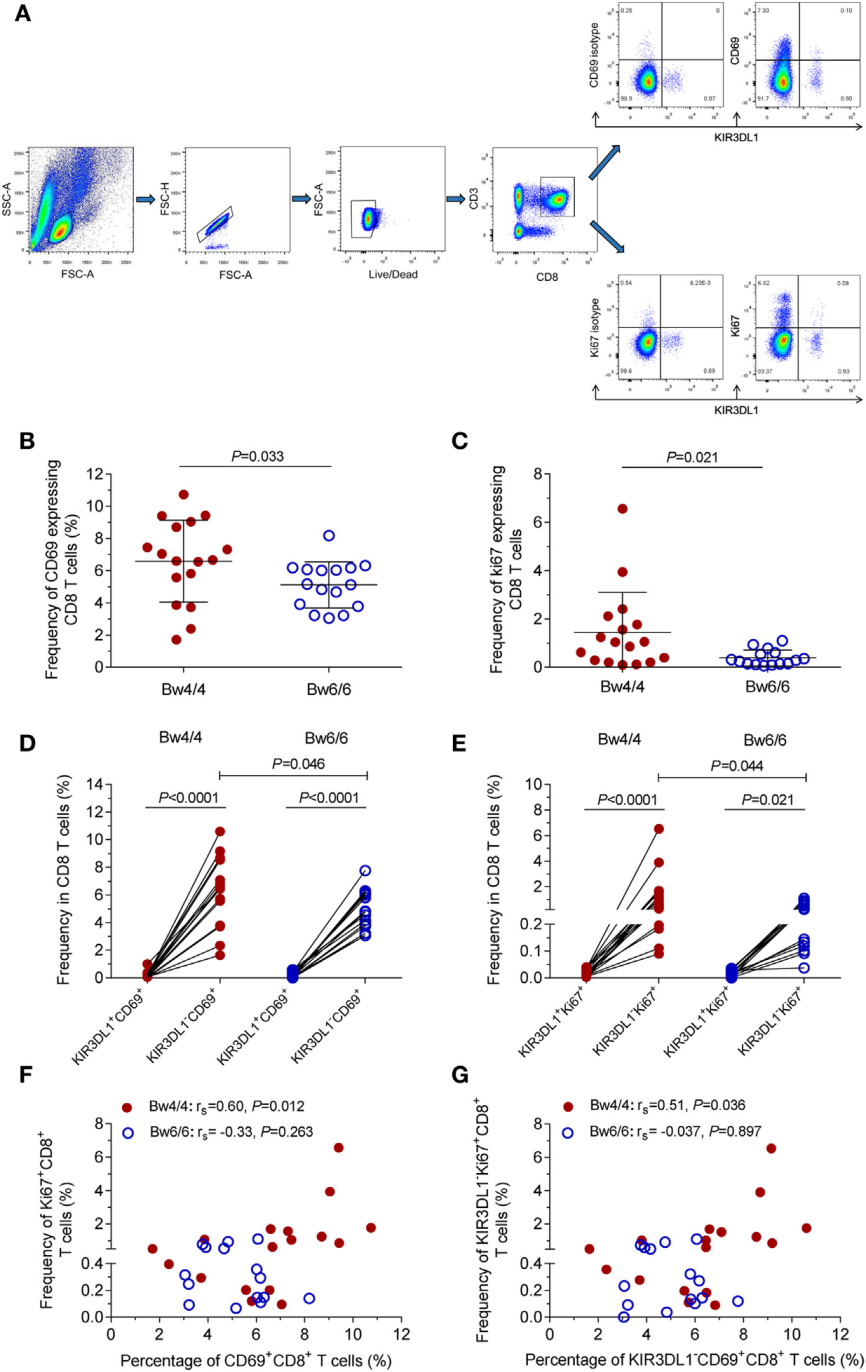
Early activation and proliferation of CD8 T cells in individuals with different *HLA-B* serological genotypes. **(A)** Gating strategy for flow cytometric analysis of CD69 and Ki67 expression on CD8 T cells; **(B)** early activation capacity of CD8 T cells; **(C)** proliferation capacity of CD8 T cells; **(D)** proportion of KIR3DL1^+^CD69^+^ cells and KIR3DL1^−^CD69^+^ cells in total CD8 T cells; **(E)** proportion of KIR3DL1^+^Ki67^+^ cells and KIR3DL1^−^Ki67^+^ cells in total CD8 T cells. Relationship between the early activation and proliferation capacity of total CD8 T cells **(F)** and KIR3DL1^−^CD8^+^ T cells **(G)**. Comparisons between two groups were performed with unpaired Student’s *t*-tests, and correlations between two variables were analyzed in Spearman’s rank correlation tests, with *P* < 0.05 considered significant.

Following infection with HIV-1, immune cells, including CD8 T cells, are activated to fight this pathogen. CD69 is one of the earliest T-cell activation markers detected, due to its rapid appearance on the surface of the plasma membrane after stimulation (32). Figure 4A displayed the flow cytometric profiles of KIR3DL1 and CD69 expression on CD8 T cells. In this study, the levels of CD69 expression on CD8 T cells during acute/early HIV-1 infection were significantly higher in *Bw4*-homozygous individuals than in *Bw6*-homozygous individuals (*P* = 0.033, Figure 4B). When individuals encounter the HIV-1, their immune cell populations, including CD8 T cells, expand to protect the host against viral infection after activation. Ki67 antigen is required for cell proliferation and used as an excellent marker of cell proliferation (33). The proliferative capacity of CD8 T cells was significantly higher in HIV-1-infected individuals homozygous for the *Bw4* allele than in those homozygous for *Bw6* (*P* = 0.021, Figure 4C). However, the levels of CD69^+^CD8^+^ T cells or Ki67^+^CD8^+^ T cells were not associated with HIV-1 VL set point or CD4 T-cell count (data not shown).

In addition, the frequency of KIR3DL1^+^CD69^+^CD8^+^ T cells was much lower than that of KIR3DL1^−^CD69^+^CD8^+^ T cells in HIV-1-infected individuals, regardless of *Bw4* or *Bw6* homozygosity (all *P* < 0.0001, Figure 4D). Nevertheless, the proportion of KIR3DL1^−^CD69^+^CD8^+^ T cells in HIV-1-infected individuals homozygous for *Bw4* was significantly higher than that in individuals homozygous for *Bw6* (*P* = 0.046, Figure 4D). The percentage of KIR3DL1^+^Ki67^+^CD8^+^ T cells was significantly lower than that of KIR3DL1^−^Ki67^+^CD8^+^ T cells in both individuals homozygous for *Bw4* and in those homozygous for *Bw6* (all *P* < 0.05, Figure 4E). By contrast, the proportion of KIR3DL1^−^Ki67^+^CD8^+^ T cells was significantly higher in individuals homozygous for *Bw4* than in those homozygous for *Bw6* (*P* = 0.044, Figure 4E). Thus, a minority of KIR3DL1^+^CD8^+^ T cells and the majority of KIR3DL1^−^CD8^+^ T cells constituted the expanded CD8 T-cell population, and this specificity was not associated with *Bw4* or *Bw6* homozygosity. Here, the levels of KIR3DL1^−^CD69^+^CD8^+^ T cells or KIR3DL1^−^Ki67^+^CD8^+^ T cells were not associated with HIV-1 VL set point or CD4 T-cell count (data not shown). Furthermore, the numbers of CD69^+^CD8^+^ T cells and KIR3DL1^−^CD69^+^CD8^+^ T cells were positively correlated with the percentages of Ki67^+^CD8^+^ T cells (*r*_s_ = 0.60, *P* = 0.012, Figure 4F) and KIR3DL1^−^Ki67^+^CD8^+^ T cells (*r*_s_ = 0.51, *P* = 0.036, Figure 4G), respectively, in individuals homozygous for *Bw4*.

### HIV-1-Specific CD8^+^ T-Cell Responses Were Stronger for KIR3DL1^−^CD8^+^ T Cells

**Figure 5:**
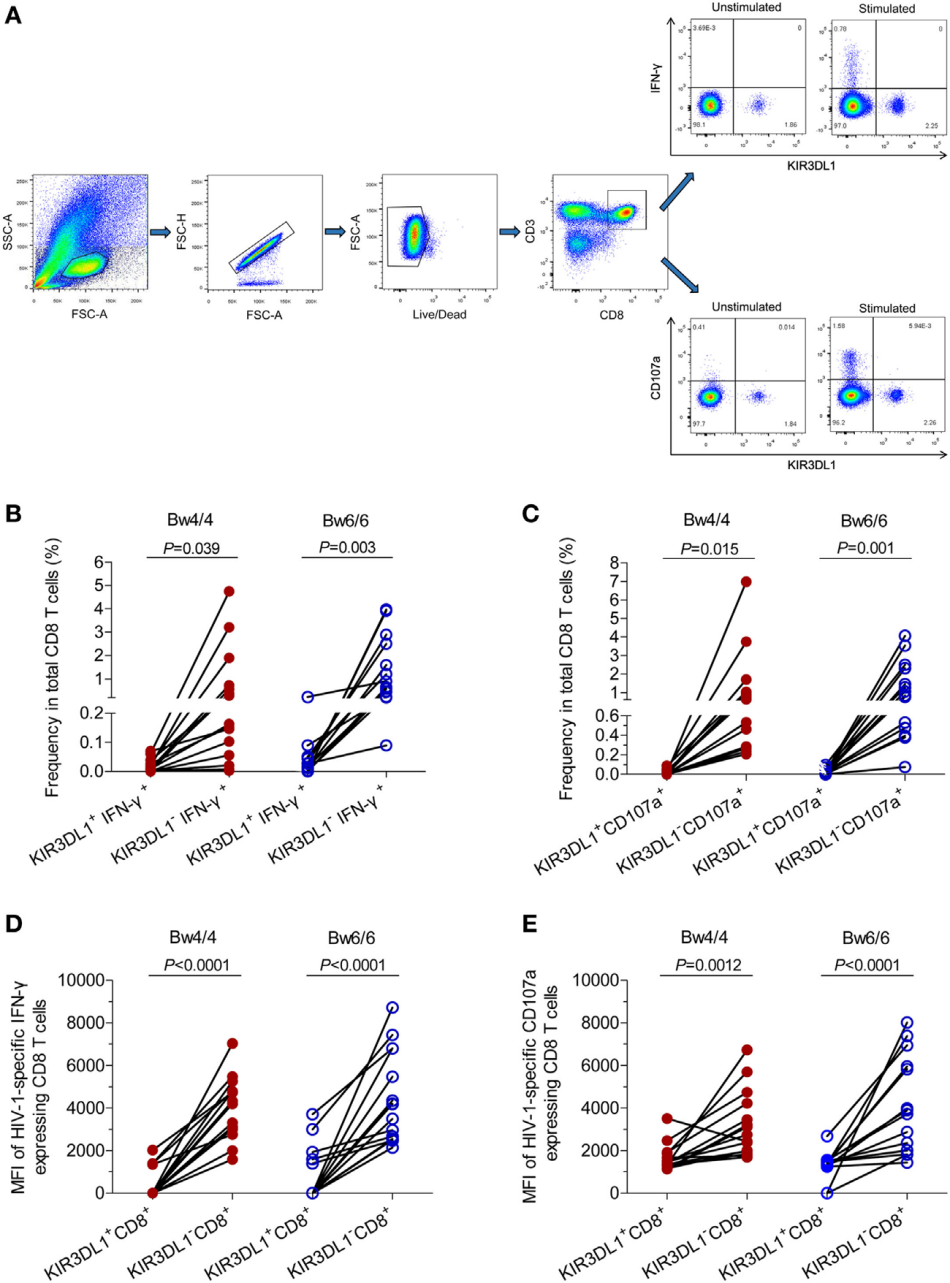
Median fluorescence intensity and levels of human immunodeficiency virus type 1 (HIV-1)-specific IFN-γ and CD107a produced by CD8 T cells, in *Bw4* and *Bw6* homozygotes. **(A)** Gating strategy for flow cytometric analysis of IFN-γ and CD107a expressing in CD8 T cells; **(B)** frequency of KIR3DL1^+^IFN-γ^+^ cells and KIR3DL1^−^IFN-γ^+^ cells among total CD8 T cells stimulated with HIV-1 p24 peptides; **(C)** frequency of KIR3DL1^+^CD107a^+^ and KIR3DL1^−^CD107a^+^ cells among total CD8 T cells stimulated with HIV-1 p24 peptides. The difference in median fluorescence intensity for the HIV-1-specific IFN-γ **(D)** and CD107a **(E)** released by KIR3DL1^+^CD8^+^ and KIR3DL1^−^CD8^+^ T cells. The Bw4/4 and Bw6/6 motifs are shown in red and blue, respectively. Paired Student’s *t*-tests were used to compare groups, with *P* < 0.05 considered significant.

We studied HIV-1-specific cytokine secretion and the degranulation of CD8 T cells in acute/early HIV-1 infection, by stimulating PBMCs with the p24 peptides pool and measuring the amounts of IFN-γ and CD107a produced by CD8 T cells (Figure 5). The flow cytometry gating strategy for the analysis of IFN-γ and CD107a secretions by CD8 T cells was displayed in Figure 5A. The levels of HIV-1-specific CD8 T cells secreting IFN-γ and CD107a were similar in individuals homozygous for *Bw4* and those homozygous for *Bw6*. The frequencies of KIR3DL1^+^IFN-γ^+^ cells and KIR3DL1^+^CD107a^+^ cells as a proportion of total CD8 T cells were lower than those of KIR3DL1^−^IFN-γ^+^ cells and KIR3DL1^−^CD107a^+^ cells, respectively, in HIV-1-infected individuals, regardless of homozygosity for *Bw4* or *Bw6* (all *P* < 0.05, Figures 5B,C). Furthermore, the fluorescence intensity obtained for IFN-γ secretion by KIR3DL1^+^CD8^+^ T cells was significantly lower than that for secretion by KIR3DL1^−^CD8^+^ T cells in these two groups of individuals (all *P* < 0.0001, Figure 5D). Likewise, the fluorescence intensity obtained for CD107a degranulation by KIR3DL1^+^CD8^+^ T cells was also significantly lower than that for KIR3DL1^−^CD8^+^ T cells (*P* = 0.0012 for *Bw4* homozygotes; *P* < 0.0001 for *Bw6* homozygotes, Figure 5E) for these two genotypes of individuals. These data suggest that KIR3DL1^−^CD8^+^ T cells play a predominant role in HIV-1-specific CD8 T-cell cytokine secretion and degranulation in acute/early HIV-1 infection, independent of *Bw4* homozygosity.

### The Levels of HIV-1-Specific KIR3DL1^−^CD8^+^ T Cells Secreting IFN-γ Were Inversely Correlated With the Early Activation of KIR3DL1^−^CD8^+^ T Cells

CD8 T cells are activated by HIV-1, leading to their production of cytokines, such as IFN-γ, to suppress virus replication. In this study, the levels of HIV-1-specific CD8 T cells secreting IFN-γ were inversely correlated with the early activation of CD8 T cells in individuals homozygous for *Bw4* (*r*_s_ = −0.81, *P* = 0.0007, Figure S3A in Supplementary Material), but not in those homozygous for *Bw6* (*r*_s_ = −0.23, *P* = 0.385, Figure S3A in Supplementary Material). Interestingly, the number of HIV-1-specific KIR3DL1^−^IFN-γ^+^CD8^+^ T cells in *Bw4*-homozygous individuals was also inversely correlated with the frequency of KIR3DL1^−^CD69^+^CD8^+^ T cells (*r*_s_ = −0.54, *P* = 0.050, Figure S3B in Supplementary Material). These findings suggest that higher levels of CD69 expression on CD8 T cells are correlated with lower levels of HIV-1-specific IFN-γ release by CD8 T cells in *Bw4* homozygotes. This was further confirmed by the observation that very few HIV-1-specific CD8^+^ T cells expressed CD69 (Figure S3C in Supplementary Material). By contrast, CD69^−^CD8^+^ T cells could be induced specifically by HIV-1 to produce specific IFN-γ and CD107a (Figure S3C in Supplementary Material).

### Similar Strength and Breadth of p24-Specific CD8 T Cell Responses Were Induced in *Bw4*- and *Bw6*-Homozygous Individuals

**Figure 6:**
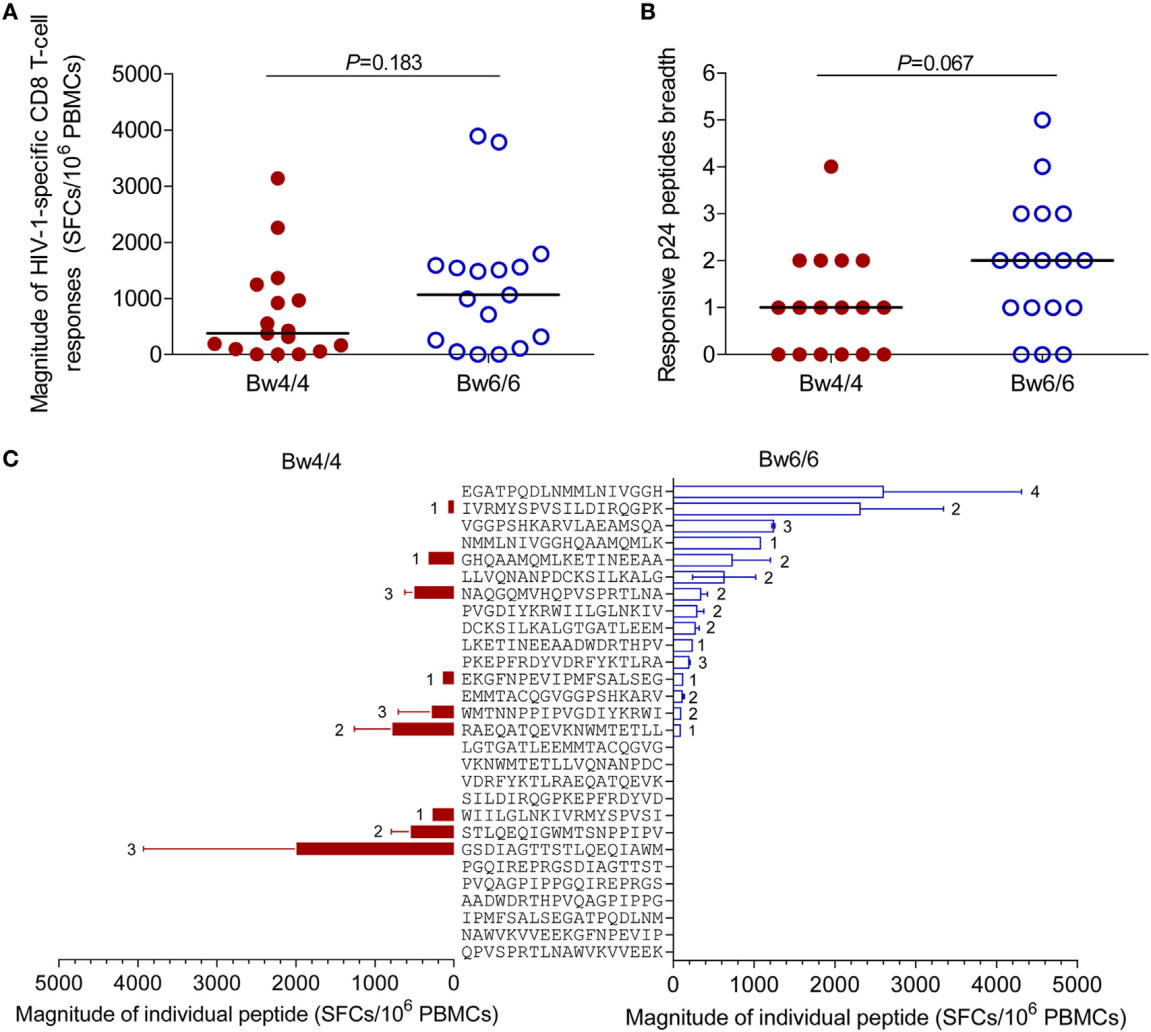
Comparison of human immunodeficiency virus type 1 (HIV-1)-specific CD8 T-cell responses. Comparison of the magnitude **(A)** and the breadth **(B)** of the p24-specific CD8 T-cell responses induced in patients homozygous for *Bw4* or *Bw6*; **(C)** Differences in peptide mapping for patients homozygous for *Bw4* (red) and *Bw6* (blue). The location of individual peptide was shown in Table S1 in Supplementary Material. The number beside the bar indicates the number of patients responding to the peptide concerned. Unpaired, non-parametric Mann–Whitney *U* tests were used to compare groups, with *P* < 0.05 considered significant.

As the levels of HIV-1-specific CD8 T cells secreting IFN-γ in *Bw4*-homozygous individuals were similar to those in *Bw6*-homozygous individuals, we investigated whether the strength and breadth of HIV-1-specific CD8 T-cell responses induced by individual p24 peptides were similar in the two groups of individuals. The median magnitude of the p24-specific CD8 T-cell responses elicited in individuals homozygous for *Bw4* was 375 (0–3,135) SFCs/10^6^ PBMCs, a value similar to the 1,065 (0–3,890) SFCs/10^6^ PBMCs in *Bw6*-homozygous individuals (*P* = 0.183, Figure 6A). Furthermore, the breadth of the CD8 T-cell responses induced by individual p24 peptides in *Bw4*-homozygous individuals was 1 (range, 0–4), a value tending toward significance to that obtained for *Bw6*-homozygous individuals (2; range: 0–5; *P* = 0.067, Figure 6B). The difference in p24 peptide mapping between individuals homozygous for *Bw4* and those homozygous for *Bw6* is shown in Figure 6C. The ELISPOT assays confirmed the similar magnitude and breadth of the HIV-1-specific CD8 T-cell responses elicited by individual p24 peptides in individuals homozygous for *Bw4* and individuals homozygous for *Bw6* in the acute/early stage of HIV-1 infection.

### KIR3DL1^−^NK Cell Activation and HIV-1-Specific KIR3DL1^−^CD8 T-Cell Responses Were Inversely Correlated With HIV-1 VL Set Point in *Bw4*-Homozygous Individuals

**Figure 7:**
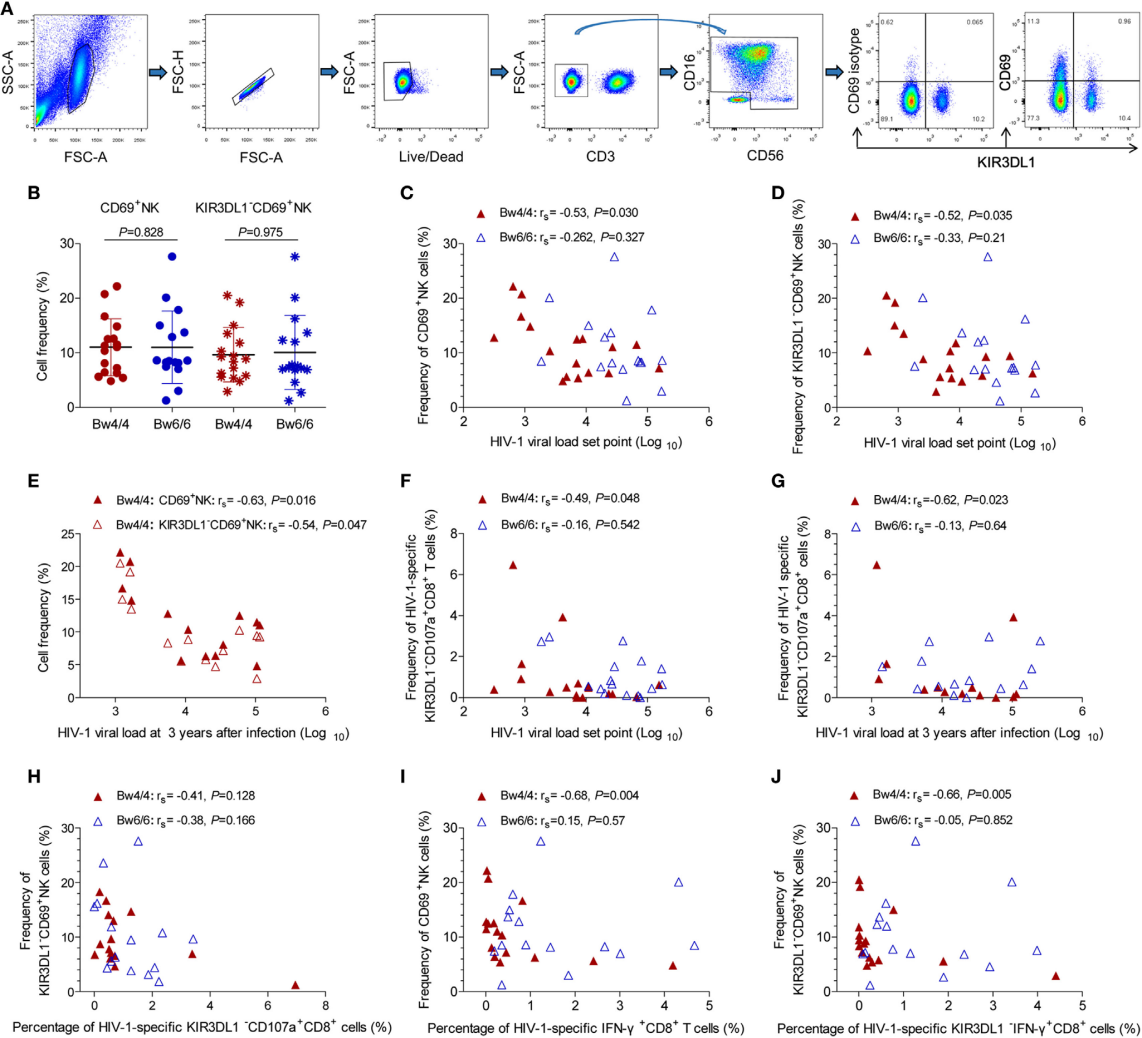
Inverse correlation between KIR3DL1^−^ natural killer (NK) cell activation or human immunodeficiency virus type 1 (HIV-1)-specific KIR3DL1^−^CD8 T-cell responses and viral load (VL) set point in *Bw4*-homozygous individuals. **(A)** Gating strategy for flow cytometric analysis of CD69 and KIR3DL1 expressing on NK cells; **(B)** comparison of the proportion of CD69^+^NK and KIR3DL1^−^CD69^+^NK cells between HIV-1-infected individuals homozygous for *Bw4* (red) and *Bw6* (blue). Inverse correlation between the frequency of CD69^+^NK cells **(C)**, KIR3DL1^−^CD69^+^NK cells **(D)** and HIV-1 VL set point, and even HIV-1 VL at 3 years after infection **(E)** in *Bw4*-homozygous individuals; negative correlation of the frequency of HIV-1-specific KIR3DL1^−^CD107a^+^CD8^+^ T cells with HIV-1 VL set point **(F)** and HIV-1 VL at 3 years after infection **(G)** in *Bw4*-homozygous participants; correlation between the KIR3DL1^−^NK cells activation capacity and the frequency of HIV-1-specific KIR3DL1^−^CD107a^+^CD8^+^ T cells **(H)**; inverse correlation between the frequency of CD69^+^NK cells and the amounts of HIV-1-specific IFN-γ^+^CD8^+^ T cells **(I)**, between the frequency of KIR3DL1^−^CD69^+^NK cells and the amounts of HIV-1-specific KIR3DL1^−^IFN-γ^+^CD8^+^ T cells **(J)** were shown. Comparisons between two groups were performed with unpaired Student’s *t*-tests and correlations between two variables were assessed in non-parametric Spearman’s rank correlation tests, with *P* < 0.05 considered significant.

As no advantageous effect of the p24-specific CD8 T-cell responses induced in *Bw4*-homozygous individuals was observed (Figures 5 and 6), we hypothesized that the inhibition of viral replication in these individuals might be due to an increase in NK cell activity. The compound expression profiles of KIR3DL1 and CD69 molecules on NK cells showed that CD69 predominantly expressed on KIR3DL1^−^NK cells (Figure 7A). The early activation levels for both total NK cells and KIR3DL1^−^NK cells did not differ between *Bw4*- and *Bw6*-homozygous individuals (*P* > 0.05, Figure 7B). However, the activation capacity of total NK cells (*r*_s_ = −0.53, *P* = 0.030, Figure 7C) and KIR3DL1^−^NK cells (*r*_s_ = −0.52, *P* = 0.035, Figure 7D) was, respectively, inversely related to HIV-1 VL set point in *Bw4*-homozygous individuals, but not in *Bw6*-homozygous individuals. Interestingly, this NK cell activation capacity was also negatively related to the levels of HIV-1 VL at 3 years after infection in *Bw4*-homozygous individuals (*r*_s_ = −0.63, *P* = 0.016 for total NK cells; *r*_s_ = −0.54, *P* = 0.047 for KIR3DL1^−^NK cells; Figure 7E), but not in *Bw6*-homozygous individuals (data not shown). These results suggest that NK cell, principally KIR3DL1^−^NK cell activation may decrease HIV-1 VL in *Bw4*-homozygous individuals.

In addition, the numbers of HIV-1-specific KIR3DL1^−^CD107a^+^CD8^+^ T cells was inversely associated with the VL set point (*r*_s_ = −0.49, *P* = 0.048, Figure 7F) and even the levels of VL at 3 years after infection (*r*_s_ = −0.62, *P* = 0.023, Figure 7G) in individuals homozygous for *Bw4*, but not for *Bw6*. These results suggest that KIR3DL1^−^CD107a^+^CD8^+^ T cells and activated KIR3DL1^−^NK cells simultaneously inhibited HIV-1 viral replication in *Bw4*-homozygous individuals, but the KIR3DL1^−^NK cell activation levels had no correlation with the numbers of HIV-1-specific KIR3DL1^−^CD107a^+^CD8^+^ T cells (*r*_s_ = −0.41, *P* = 0.128, Figure 7H) in *Bw4*-homozygous individuals.

Interestingly, total NK cell activation capacity was negatively related to the numbers of HIV-1-specific IFN-γ^+^CD8^+^ T cells (*r*_s_ = −0.68, *P* = 0.004, Figure 7I) in *Bw4*-homozygous individuals. Likewise, this inverse relationship was exhibited between KIR3DL1^−^NK cell activation capacity and the amounts of HIV-1-specific KIR3DL1^−^IFN-γ^+^CD8^+^ T cells (*r*_s_ = −0.66, *P* = 0.005, Figure 7J). Thus, strong KIR3DL1^−^NK cell activation capacity, which is related to the control of HIV-1 disease progression, was associated with the weak HIV-1-specific CD8 T-cell responses in *Bw4*-homozygous individuals from our Beijing PRIMO Cohort.

## Discussion

In this study, KIR3DL1-positive CD8 T cells, was not related to the percentage of NK cells expressing KIR3DL1, in either HIV-1-positive or HIV-1-negative individuals, indicating the presence of different pathways regulating KIR3DL1 expression on NK and CD8 T cells. KIR3DL1-positive CD8 T cells did not increase in acute/early HIV-1 infection (Figure 2), whereas the KIRs-positive CD8 T cells, including KIR3DL1, has been shown to increase in untreated individuals with chronic HIV-1 infection (17). The higher proportion of KIR3DL1-expressing CD8 T_EMRA_ cells in *Bw6*-homozygous individuals than in *Bw4*-homozygous individuals suggested that the weak antiviral activity observed in individuals homozygous for *Bw6* was due to 76.5 (71.5–92.6%) of KIR3DL1^+^CD8^+^ T cells expressing CD57 (19), a marker of cell immunosenescence.

Indeed, the early activation and proliferation of KIR3DL1^+^CD8^+^ T cells were very weak, and the levels of HIV-1-specific KIR3DL1-expressing CD8 T cells secreting IFN-γ and expressing CD107a were very low, regardless of whether the individuals was homozygous for *Bw4* or *Bw6* (Figures 4 and 5). Similarly, several studies have demonstrated that most HIV-1-specific CD8 T cells lack KIR expression (17, 19). Together, these results suggest that the KIR3DL1^+^CD8^+^ T cells do not play a crucial role in controlling HIV-1 infection and these CD8 T cells are not responsible for the beneficial effects observed in *Bw4* homozygotes.

*HLA-B Bw4*-homozygous individuals displayed stronger CD8 T-cell early activation and proliferation, particularly for KIR3DL1^−^CD8^+^ T cells, than *Bw6* homozygotes (Figure 4), suggesting that the favorable effects of *Bw4* homozygosity are associated with KIR3DL1-negative CD8 T cells. Surprisingly, the intracellular cytokine staining and ELISPOT assays showed that the HIV-1-specific CD8 T-cell responses induced in *HLA-B Bw4*-homozygous individuals were no stronger than those induced in *Bw6*-homozygous individuals (Figure 6). As shown in Figure 6C and Table S1 in Supplementary Material, several peptides were recognized by both *Bw4* and *Bw6*-homozygous individuals, but others were only recognized and induced stronger responses in individuals homozygous for *Bw4* (gag_233–250_, which contained the CTL epitope TSTLQEQIAW restricted by B*57 and B*58) or *Bw6* (gag_177–194_, which contained the CTL epitope TPQDLNMMLN restricted by B*07 and B*42). Nonetheless, the overall p24-specific responses did not differ between these two groups (Figure 6C). Our findings were inconsistent with other studies reporting the elicitation of strong HIV-1-specific CD8 T-cell responses in HLA-B*57 and/or-B*27 individuals (34, 35). It would have been desirable to compare the HIV-1-specific responses induced on *Bw4*-homozygous individuals harboring HLA-B*27 and/or -B*57 individuals to those homozygous for *Bw6* in this study. Unfortunately, only three individuals carried HLA-B*27 and/or -B*57 alleles (Table 1). Indeed, HLA-B*57 is rare in the Chinese population (10, 12). Another reason may relate to the HLA-B*27 allele, which has been reported to prevent disease progression only for late-stage disease and was not linked to a strong CD8 T-cell antiviral response (36, 37). The individuals enrolled in this study were in the acute/early stage of HIV-1 infection. Moreover, in this study, isolated CD8^+^ T cells induced about 95% of the p24-specific T-cell responses (Figure 6; Table S1 in Supplementary Material), but isolated CD4^+^ T cells induced very little of the p24-specific T-cell responses recognized against the 18 amino acids length peptides pool (data not shown) in ELISPOT assay. Given the magnitude and breadth of HIV-1-specific CD8 T-cell responses was not stronger in *Bw4*-homozygous individuals than in *Bw6*-homozygous individuals, so the polyfunctionality, functional avidity, and cross-reactivity to epitope variants (38, 39) of HIV-1-specific CD8 T-cell responses requires further investigation to account for the advantageous effect of *Bw4* homozygosity in *HLA-B* alleles.

Interestingly, the effector responses of HIV-1-specific CD8 T cells particularly KIR3DL1^−^CD8^+^ T cells secreting IFN-γ were not increased in *Bw4*-homozygous individuals, but this effector responses were inversely correlated with early activation (Figure S2 in Supplementary Material). Indeed, early activated CD8 T cells produce very little IFN-γ and CD107a specifically in response to HIV-1, but CD69-positive CD8 cells in tissue can secrete other chemokines, such as transforming growth factor-β and IL-10 (32, 40, 41), to regulate immune responses. Besides, CD69 is involved in early events of lymphocyte activation, and plays a functional role in the redirected lysis mediated by activated NK cells, and we found that the level of CD69 expression on NK cells in *Bw4*-homozygous individuals was inversely correlated with HIV-1 VL set point (Figure 7). HIV-1 VL set point reflects the equilibrium between HIV-1 replication level and efficacy of immunologic response and has long been used as a prognostic marker of disease progression (42, 43). The early activation of NK cells particularly KIR3DL1^−^NK cells therefore appeared to be beneficial for HIV-1 viremia control especially in individuals homozygous for *Bw4*. An inverse relationship was observed between KIR3DL1^−^NK cell activation and HIV-1-specific KIR3DL1^−^CD8 T-cell responses, especially the production of IFN-γ (Figure 6), consistent with the findings of Tomescu’s study (44). Strong NK cell responses are associated with protective KIR3DL1*h/*y receptor and *HLA-I* allele (such as HLA-B*57), independently of the lack of increase in HIV-1 gag-specific T-cell responses in HIV-1-infected elite controllers. Waggoner et al. described a role for NK cells in the inverse modulation of antiviral CD8 T cells (45). Our findings indicate that KIR3DL1^−^NK activation was inversely related to HIV-1-specific KIR3DL1^−^CD8^+^ T-cell responses in *Bw4*-homozygous individuals, suggesting higher levels of activated KIR3DL1^−^NK cells might decrease the levels of HIV-1-specific KIR3DL1^−^CD8^+^ T cell responses, but all these cells were potent in controlling HIV-1 infection.

Kim et al. reported that KIR3DL1^+^NK cells in *HLA-Bw4* homozygous healthy individuals were more responsive to autologous target cells than in *HLA-Bw6* homozygous healthy donors (46), while the functionality of KIR3DL1^+^NK cells did not differ in *HLA-Bw4* carriers and *HLA-Bw6* homozygous individuals in HIV-1 infection (47). No differences in NK cell functionality of mediating anti-HIV ADCC responses was observed between KIR3DL1^+^NK cells and KIR3DL1^−^NK cells in *HLA-Bw4* individuals infected with HIV-1 (47), though KIR3DL1^+^NK cells from *HLA-Bw4* healthy controls were more functional than KIR3DL1^−^NK cells (46). These data suggested the functionality of KIR3DL1^+^NK cells could be attenuated due to HIV-1 infection in *HLA-Bw4* homozygous individuals. NK cells from HIV-1-uninfected *Bw6*-homozygous individuals inhibited HIV-1 replication in infected autologous CD4 cells less potently than those from protective *KIR/HLA* genotypes including KIR3DL1*h/*y and B*57 combined genotypes (48), which secreted higher levels of CC-chemokines (CCL-3 and CCL-4). Based on these reports and the outcomes of the vast majority of activated NK cells were KIR3DL1-negative NK cells, and the negative relationship between KIR3DL1^−^NK cell activation and VL set point in *Bw4*-homozygous individuals, we speculate that KIR3DL1^−^NK cells may be more active in HIV-1-infected individuals homozygous for the *Bw4* allele, although no increase in HIV-1-specific CD8 T-cell responses was observed in this study. In addition, the higher frequency of early activated and proliferated CD8 T cells, and particularly of KIR3DL1-negative CD8 T cells in *Bw4*-homozygous individuals, would have greater antiviral potential during acute/early HIV-1 infection.

**Figure 8:**
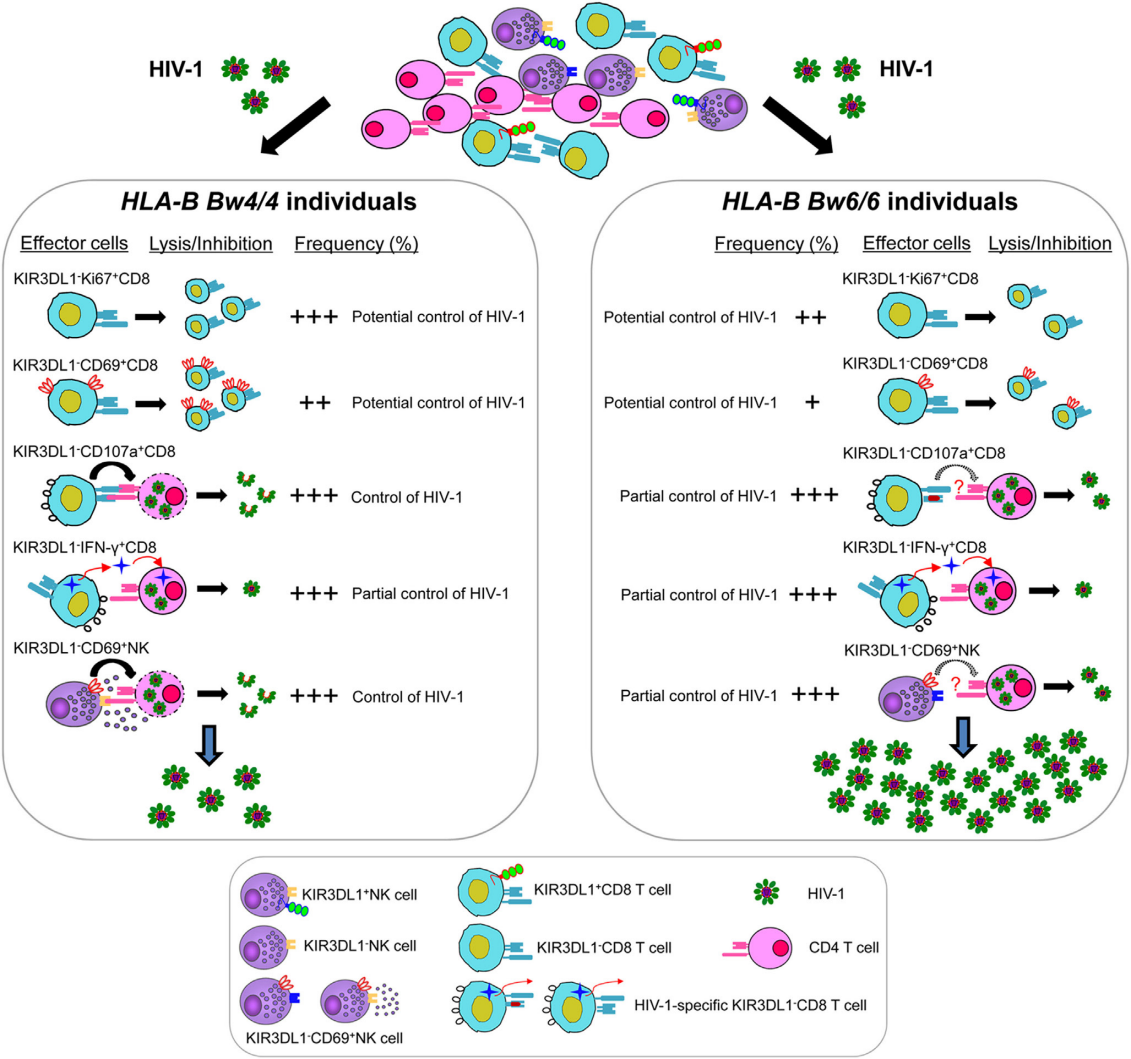
Model for KIR3DL1^−^CD8^+^ T cell and KIR3DL1^−^ natural killer (NK) cell controlling human immunodeficiency virus type 1 (HIV-1) infection in individuals with distinct *HLA-B* serological genotypes. HIV-1-specific KIR3DL1^−^CD107a^+^CD8^+^ T cells and KIR3DL1^−^CD69^+^NK cells were associated with inhibiting HIV-1 replication in *Bw4*-homozygous individuals, but not efficient in *Bw6*-homozygous individuals. The levels of HIV-1-specific KIR3DL1^−^IFN-γ^+^CD8^+^ T cells were similar in both individuals homozygous for *Bw4* and *Bw6*, may be associated with partial control of HIV-1 infection. Moreover, the proliferative capacity of KIR3DL1^−^Ki67^+^CD8^+^ T and the early activation of KIR3DL1^−^CD69^+^CD8^+^ T cells, which can potential control of HIV-1 infection, were significantly higher in HIV-1-infected individuals homozygous for the *Bw4* than in those homozygous for *Bw6*. Nevertheless, the frequency and function of KIR3DL1-expressing CD8^+^ T/NK cells was much lower than that of KIR3DL1-negative CD8^+^ T/NK cells in HIV-1-infected individuals, regardless of *Bw4* or *Bw6* homozygosity. Thus, KIR3DL1-expressing CD8^+^ T/NK cells, which associated with the loss of multiple functions, may do not play a crucial role in controlling HIV-1 infection in the early stages of HIV-1 infection.

In general, KIR3DL1 expression on CD8 T cells was associated with the loss of multiple functions, including the limitation of viral replication and the slowing of CD4 decline, early activation, proliferation, HIV-1-specific cytokine secretion, and degranulation *in vitro*. KIR3DL1^−^CD8^+^ T cells and KIR3DL1^−^NK cells in individuals homozygous for *Bw4* were related to inhibiting HIV-1 infection as summarized in Figure 8, but the underlying mechanisms are not fully clear. Thus, further studies are required to determine whether other inhibitory KIRs and other immune inhibitory receptors containing the ITIMs such as PD-1 and TIGIT (49, 50) are involved in regulating KIR3DL1^−^CD8^+^ T-cell and KIR3DL1^−^NK cell functions. Furthermore, only 34 participants infected with HIV-1 CRF01_A/E subtype were enrolled in this current study, because CRF01_A/E subtype is the most dominant strain in most regions of China. Thus, it will be worthwhile for further studies to investigate the mechanisms of advantageous effects of *Bw4* homozygosity based on other HIV-1 subtypes, and a larger number of individuals with *HLA-B Bw4* homozygotes will be also required.

Taken together, our findings demonstrate for the first time that the KIR3DL1^−^CD8 T/NK cell functions of *Bw4* homozygotes are associated with the control of early HIV-1 replication in the absence of antiretroviral treatment. These results open up new insights into the design of an effective vaccine against HIV.

## Ethics Statement

This study and all the relevant experiments were approved by the Beijing You’an Hospital Research Ethics Committee, and written informed consent was obtained from each participant in accordance with the Declaration of Helsinki. All participants provided written informed consent for the collection of information, and their clinical samples were stored and used for research. The methods used conformed to approved guidelines and regulations.

## Author Contributions

XZ, HW, TZ, and BS conceived and designed the experiments; WX, and YZ collected the sample information, contributed to reagents and materials; XZ, XL, LY, ZLi, and ZLiu performed the experiments; XZ, CM, HW, TZ, and BS analyzed the data; and XZ, CM, and BS wrote the manuscript. All authors read and approved the final manuscript.

## Conflict of Interest Statement

The authors declare that the research was conducted in the absence of any commercial or financial relationships that could be construed as a potential conflict of interest.

## Supplementary Material

Figure S1 Flow chart of human immunodeficiency virus type 1 (HIV-1) participants at enrollment. Screening and follow-up of participants in the acute/early phase of HIV-1 infection. In total, 17 of 24 participants homozygous for *Bw4* and 17 of 40 participants homozygous for *Bw6* were enrolled in this study. These 34 participants without antiretroviral therapy were infected with a CRF01_A/E subtype based on *pol* sequence and between the Fiebig stage VI and 6 months after infection. The opportunistic infections, tuberculosis, autoimmune diseases, or HBV/HCV co-infection were excluded.

Figure S2 ELISOPT assays. Representative experiments showing peripheral blood mononuclear cells from eight individuals infected with human immunodeficiency virus type 1 subtype CRF01_A/E were stimulated with CRF01_A/E p24 peptides pool. Phytohemagglutinin (PHA) was used as experimental positive control and EBV/Flu/CMV (EFC) peptides were used as quality control; negative control was used with RPMI 1640 medium.

Figure S3 Relationship between the early activation capacity of CD8 T cells and the levels of human immunodeficiency virus type 1 (HIV-1)-specific IFN-γ or CD107a released by CD8 T cells. Inverse relationship between early activation capacity and levels of HIV-1-specific IFN-γ-secreting total CD8 T cells **(A)**, and **(B)** KIR3DL1-negative CD8 T cells; **(C)** gating strategy for flow cytometric analysis of CD69, IFN-γ and CD107a combined expressing on CD8 T cells after stimulation with p24 peptides. Graphs are shown for gating on the CD8 T-cell population. Correlation between two variables was analyzed with Spearman’s rank correlation tests, with *P* < 0.05 considered significant.
