# Supplementary figures and images for: KIR3DL1-Negative CD8 T Cells and KIR3DL1-Negative Natural Killer Cells Contribute to the Advantageous Control of Early Human Immunodeficiency Virus Type 1 Infection in HLA-B Bw4 Homozygous Individuals
Source: Front Immunol. 2018 Aug 10;9:1855. doi: 10.3389/fimmu.2018.01855 (PMC6096002; doi:10.3389/fimmu.2018.01855)

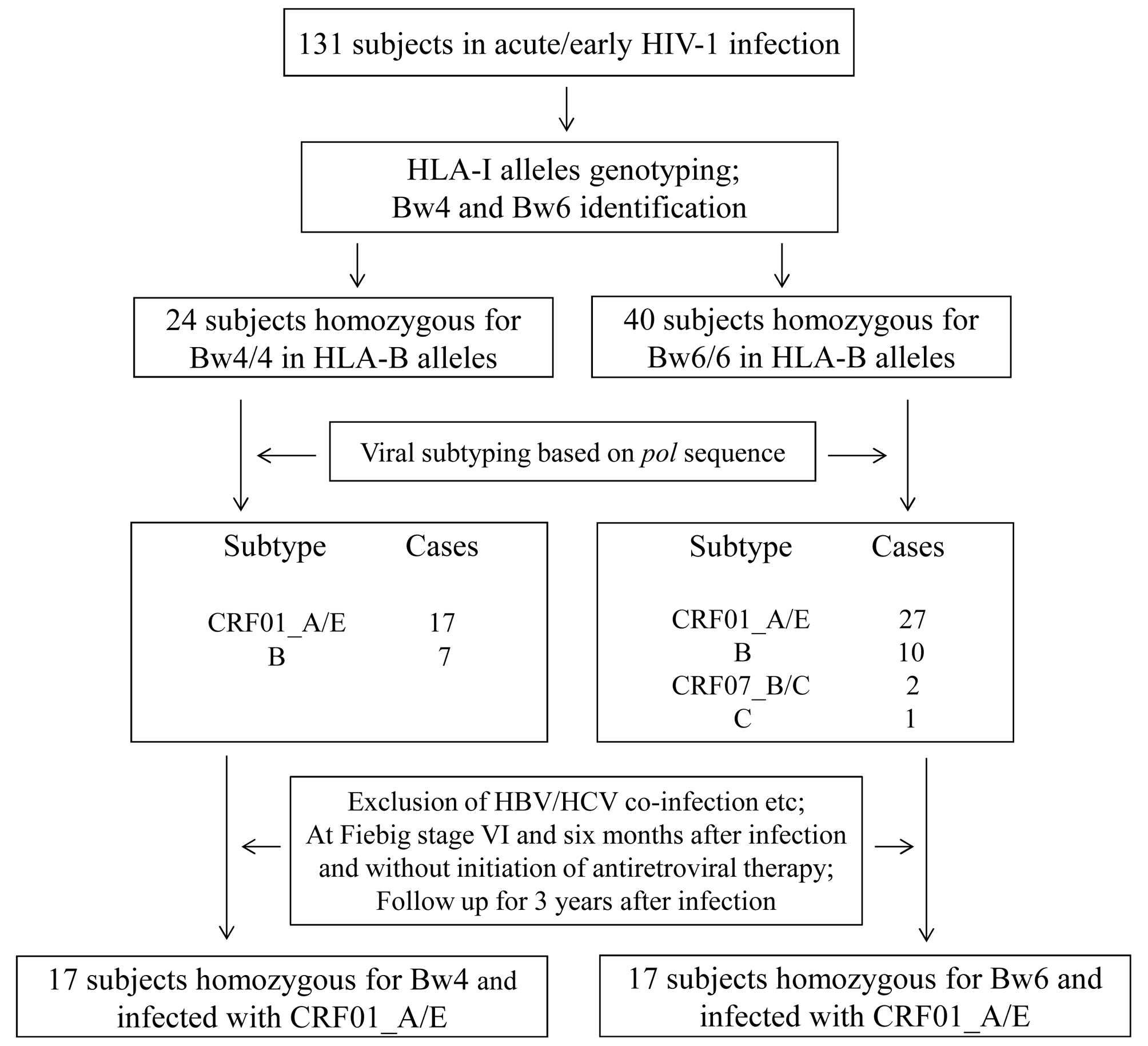

Supplement: Figure S1 — Flow chart of human immunodeficiency virus type 1 (HIV-1) participants at enrollment. Screening and follow-up of participants in the acute/early phase of HIV-1 infection. In total, 17 of 24 participants homozygous for Bw4 and 17 of 40 participants homozygous for Bw6 were enrolled in this study. These 34 participants without antiretroviral therapy were infected with a CRF01_A/E subtype based on pol sequence and between the Fiebig stage VI and 6 months after infection. The opportunistic infections, tuberculosis, autoimmune diseases, or HBV/HCV co-infection were excluded. [file image_1.tif]

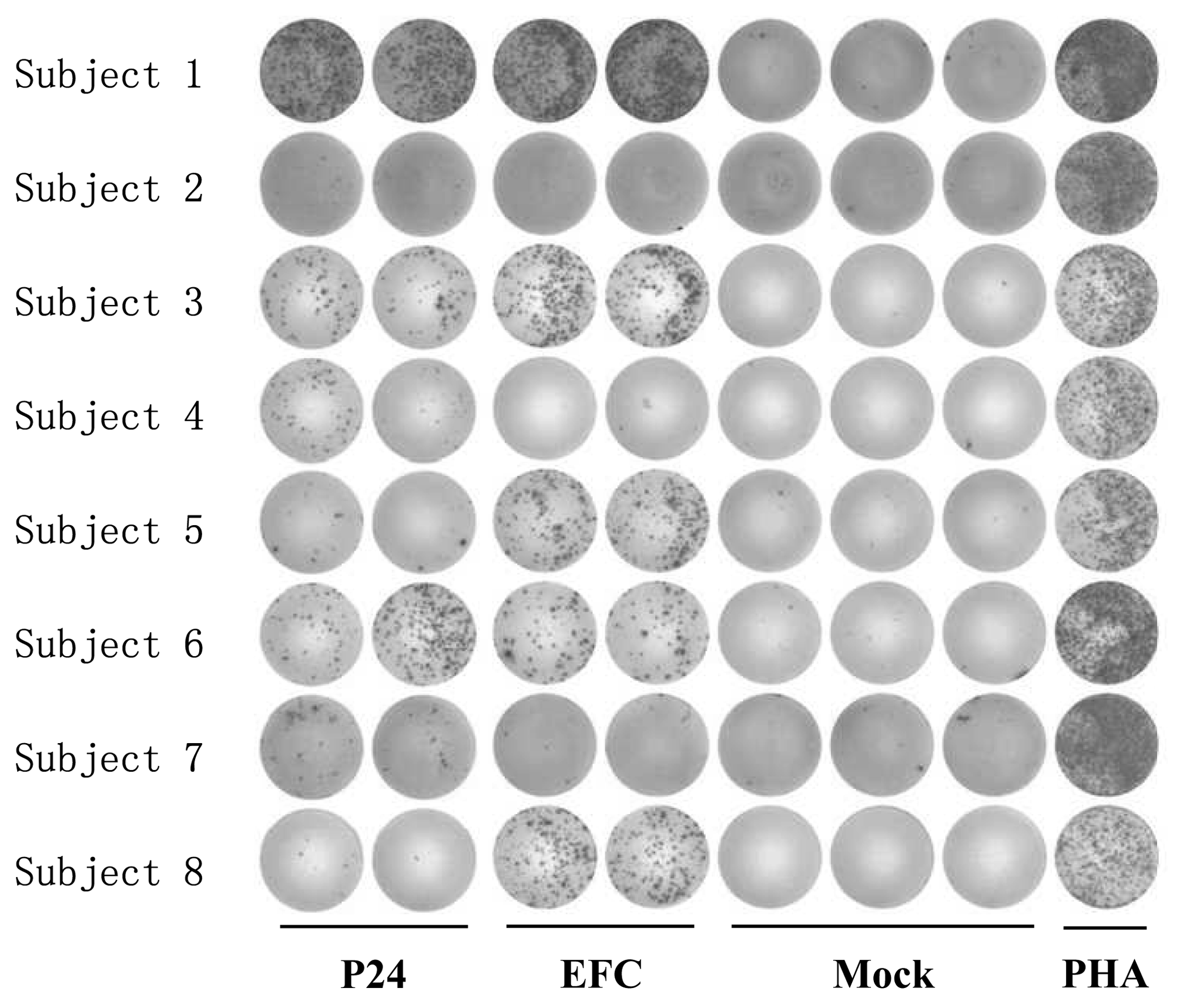

Supplement: Figure S2 — ELISOPT assays. Representative experiments showing peripheral blood mononuclear cells from eight individuals infected with human immunodeficiency virus type 1 subtype CRF01_A/E were stimulated with CRF01_A/E p24 peptides pool. Phytohemagglutinin (PHA) was used as experimental positive control and EBV/Flu/CMV (EFC) peptides were used as quality control; negative control was used with RPMI 1640 medium. [file image_2.tif]

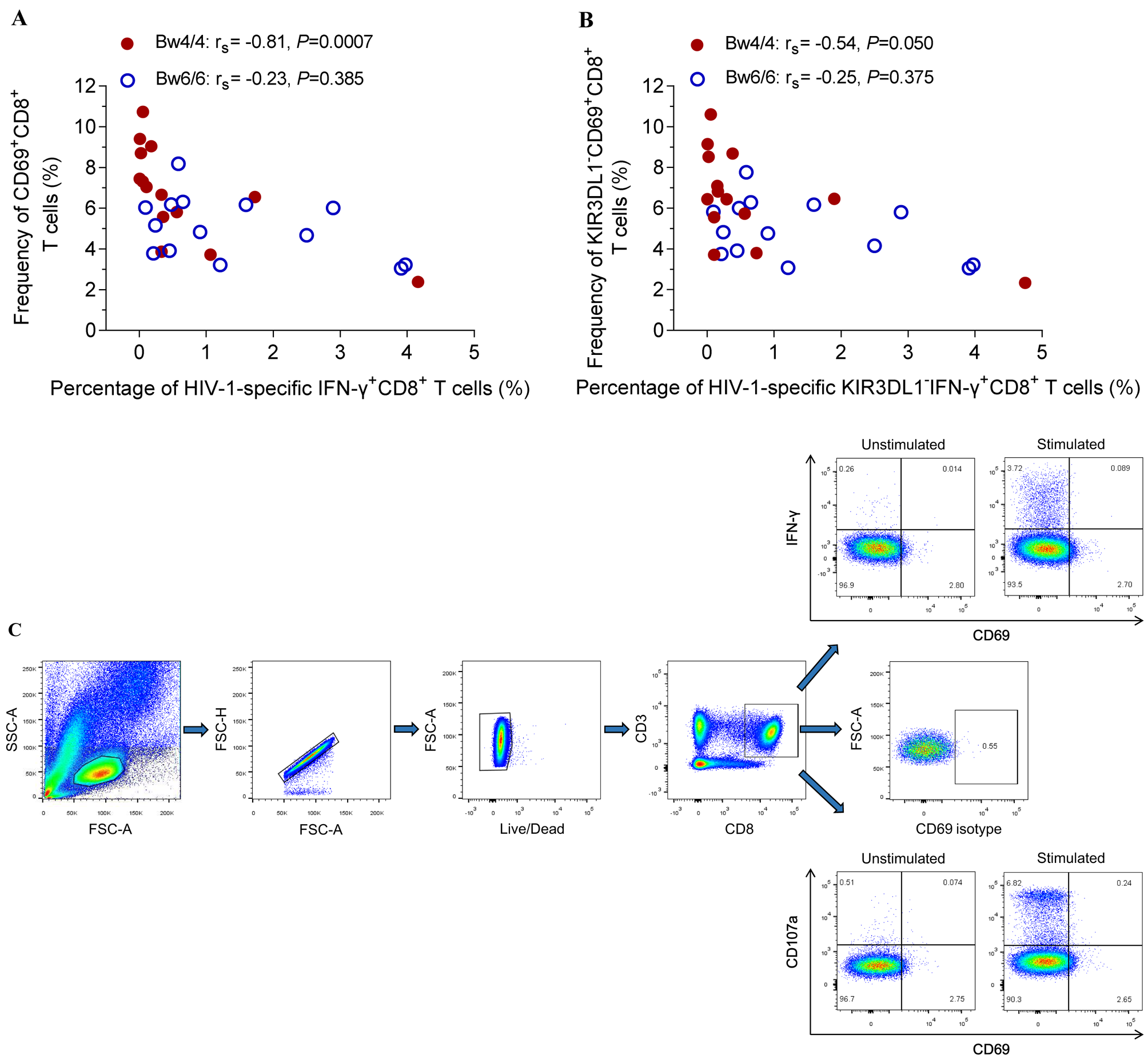

Supplement: Figure S3 — Relationship between the early activation capacity of CD8 T cells and the levels of human immunodeficiency virus type 1 (HIV-1)-specific IFN-γ or CD107a released by CD8 T cells. Inverse relationship between early activation capacity and levels of HIV-1-specific IFN-γ-secreting total CD8 T cells (A), and (B) KIR3DL1-negative CD8 T cells; (C) gating strategy for flow cytometric analysis of CD69, IFN-γ and CD107a combined expressing on CD8 T cells after stimulation with p24 peptides. Graphs are shown for gating on the CD8 T-cell population. Correlation between two variables was analyzed with Spearman’s rank correlation tests, with P < 0.05 considered significant. [file image_3.tif]
